# The Inhibitory Properties of a Novel, Selective LMTK3 Kinase Inhibitor

**DOI:** 10.3390/ijms24010865

**Published:** 2023-01-03

**Authors:** Alessandro Agnarelli, Andrea Lauer Betrán, Athanasios Papakyriakou, Viviana Vella, Mark Samuels, Panagiotis Papanastasopoulos, Christina Giamas, Erika J. Mancini, Justin Stebbing, John Spencer, Chiara Cilibrasi, Angeliki Ditsiou, Georgios Giamas

**Affiliations:** 1 Department of Biochemistry and Biomedicine, School of Life Sciences, University of Sussex, Brighton BN1 9QG, UK; 2 Institute of Biosciences and Applications, National Centre for Scientific Research “Demokritos”, 15341 Athens, Greece; 3 Department of Surgery and Cancer, Imperial College, London SW7 2BX, UK; 4 Sussex Drug Discovery Centre, School of Life Sciences, University of Sussex, Brighton BN1 9QG, UK

**Keywords:** LMTK3, kinase inhibitor, breast cancer

## Abstract

Recently, the oncogenic role of lemur tyrosine kinase 3 (LMTK3) has been well established in different tumor types, highlighting it as a viable therapeutic target. In the present study, using in vitro and cell-based assays coupled with biophysical analyses, we identify a highly selective small molecule LMTK3 inhibitor, namely C36. Biochemical/biophysical and cellular studies revealed that C36 displays a high in vitro selectivity profile and provides notable therapeutic effect when tested in the National Cancer Institute (NCI)-60 cancer cell line panel. We also report the binding affinity between LMTK3 and C36 as demonstrated via microscale thermophoresis (MST). In addition, C36 exhibits a mixed-type inhibition against LMTK3, consistent with the inhibitor overlapping with both the adenosine 5′-triphosphate (ATP)- and substrate-binding sites. Treatment of different breast cancer cell lines with C36 led to decreased proliferation and increased apoptosis, further reinforcing the prospective value of LMTK3 inhibitors for cancer therapy.

## 1. Introduction

Protein kinases are a large family of enzymes responsible for catalyzing protein phosphorylation. They are involved in critical mechanisms regulating different cellular functions, including proliferation, cell cycle, apoptosis, motility, growth, and differentiation [1]. The deregulation of protein kinase activity contributes to various human diseases and disorders, including cancer [2]. Therefore, it is not surprising that the kinome is considered an attractive target for the treatment of several tumors, leading to a shift in the clinical management of cancer and improved patient outcome [3]. However, despite promising results, the inevitable development of drug resistance, largely due to the activation of complementary and/or compensatory pathways, remains a major limitation for this therapeutic approach [4,5].

Lemur tyrosine kinase 3 (LMTK3) is a dual specificity serine/threonine kinase composed of a transmembrane helical segment, a kinase domain, and a C-terminal intrinsically disordered region [6]. Studies have put forward a physiological role for LMTK3 in neuron trafficking where LMTK3 knockout can cause behavioral abnormalities in mice [7]. Although information regarding the function of LMTK3 in normal physiology is limited, its oncogenic role has been well established so far in various tumor types, including bladder, lung, and colorectal cancer, among others, highlighting it as a potential therapeutic target [8,9,10,11,12,13,14,15,16,17,18,19,20,21,22]. LMTK3 was originally identified as an important regulator of estrogen receptor alpha (ERα) activity in breast cancer (BC) following a whole human kinome siRNA screen [8]. Specifically, LMTK3 was shown to directly protect ERα from ubiquitin-mediated proteasomal degradation and indirectly promote ERα transcription through the PKC/AKT signaling pathway [8]. Follow-up studies have further supported that elevated levels of LMTK3 in BC are associated with poorer overall survival (OS) and disease-free survival (DFS) [12]. Moreover, LMTK3 has also been implicated in endocrine [13] and chemotherapy resistance in BC [14], while us and others have described an involvement of LMTK3 in different signaling pathways [13,23].

Recently, using robust in vitro and cell-based screening and selectivity assays combined with biophysical analyses, we identified and characterized a highly selective small-molecule adenosine 5′-triphosphate (ATP)-competitive LMTK3 inhibitor, namely C28, that acts by degrading LMTK3 via the ubiquitin-proteasome pathway [2]. Overall, C28 exhibited effective anticancer effects in several cancer cell lines, as well as in vivo BC mouse models (xenograft and transgenic) [2]. Here, we report the inhibitory properties of another compound (C36) against LMTK3, further supporting the rationale that the development and optimization of LMTK3 inhibitors can have prospective value to cancer patients.

## 2. Results

### 2.1. Selectivity Profile of C36 Inhibitor

**Figure 1:**
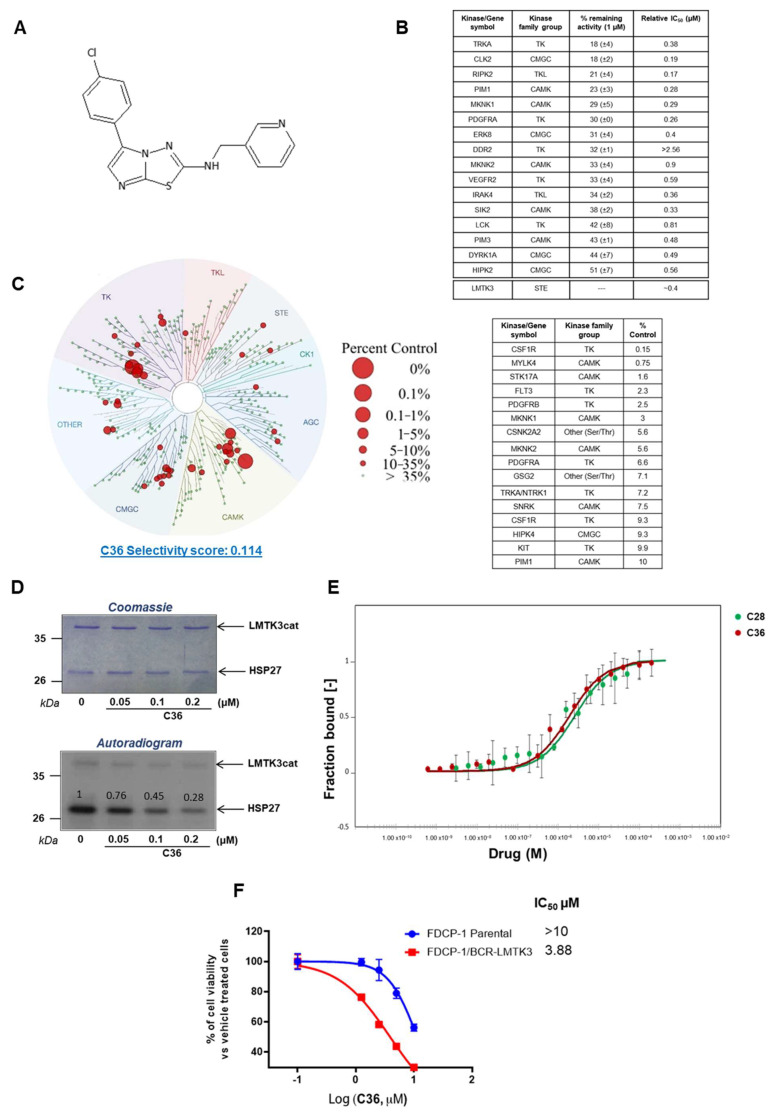
Selectivity of C36 toward LMTK3. (**A**) Chemical structure of C36. (**B**) Selectivity profile of C36 (1 µM) against 140 kinases using radioactive filter binding assay (MRC International Centre for Kinase Profiling unit). The data are displayed as the percentage of activity remaining of assay duplicates with a SD. Only kinases with >50% decrease in their activity are shown. The relative IC_50_ values are also presented. (**C**) TREEspot interaction map depicting the kinome phylogenetic grouping, with kinases interacting with C36 (5 µM) represented as red circles (DiscoverX KINOMEscan [25]). Kinases whose binding affinity was inhibited by C36 to less than 10% of the control (DMSO) are shown in the table. Lower numbers indicate the most probable hits to bind to C36. The larger the diameter of the circle, the higher the C36 binding affinity to the respective kinase active site. (**D**) The IC_50_ value for C36 against LMTK3cat (LMTK3 kinase domain) was determined by in vitro kinase assay. The intensities of the bands on the autoradiogram have been quantified using ImageJ software and normalized to total protein levels based on Coomassie Blue stained membranes. DMSO has been used as a control (**E**) MST binding curves for C36 (K_d_ = 1.87 ± 0.2 µM, red curve) and C28 (K_d_ = 2.50 ± 0.4 µM, green curve) with LMTK3, showing fraction bound on the Y axis and drug concentration (M) on the X axis. More specifically, fraction bound is calculated as the ratio between the emitted fluorescence of LMTK3-C36/C28 complex and the curve amplitude [26]. The error bars represent the SD of each data point calculated from three independent experiments. Binding check analysis reveals no interaction between DMSO (control) and LMTK3 kinase domain (signal to noise ratio: 1.2) (Figure S2). (**F**) IC_50_ values for C36 in FDCP1 and FDCP1-LMTK3 cell lines. Error bars represent the means ± SD from three independent experiments.

Considering the oncogenic role of LMTK3, a library encompassing 28,716 compounds (Charles River Discovery Research Services, Chesterford Research Park, UK Ltd.; formerly known as BioFocus DPI Ltd.) was screened using robust in vitro and cell-based assays identifying a potent small-molecule ATP-competitive LMTK3 inhibitor (C28), as previously described [2]. Among the hit compounds that were identified, C36 also emerged as a potential selective LMTK3 inhibitor (Figure 1A).

To obtain a more detailed analysis of the selectivity profile of C36, we performed a radioactive filter binding assay, screening this inhibitor against a series of 140 kinases [24]. Our results identified 16 kinases whose activity was reduced by >50% in the presence of 1 µM C36 (Figure 1B) compared to 18 kinases when using C28 as previously described [2].

To further examine the specificity of C36, we used an active site-directed competition binding assay (DiscoverX KINOMEscan, San Diego, CA, USA [25]) and quantitatively measured the interactions between C36 and 403 purified human kinases. Figure 1C represents a TREEspot interaction map of our compound against 403 kinases. C36 was tested at a 5 µM final concentration and the red circles indicate kinases to which C36 binds at their active site at this concentration. In addition, the size of the circle is also proportional to the binding affinity of C36 to the respective kinase (i.e., the larger the diameter of the circle, the higher the binding affinity of C36). The data shown in the table (Figure 1C) display the most significant hits from the TREEspot interaction map. The percentage of DMSO (control) is also indicated, with 10% being the highest amount used. In particular, the lower the numbers in the “% control” column, the more probable C36 binds the kinase active site. The S(35) selectivity index of C36 was 0.114, as measured by the percentage of the kinome inhibited below 35% of the control at this concentration using the following equation:

(1) $$\;S\left(35\right)=\frac{\mathrm{number}\;\mathrm{of}\;\mathrm{kinase}\;\mathrm{with}\;\%\;\mathrm{Ctrl}<35}{\mathrm{number}\;\mathrm{of}\;\mathrm{kinases}\;\mathrm{tested}}$$

Interestingly, the selectivity score for C36 (0.114) was lower when compared to C28 (0.186) [2], indicating a higher in vitro selectivity of C36 versus C28. More specifically, C36 inhibited the activity of 16 out of a total of 403 kinases by >90% (Figure 1C) compared to 33 out of 403 for C28 [2], with 5 of them overlapping (MYLK4, FLT3, GSG2, TRKA, HIPK4). The selectivity profile of C36 was determined using assays with different underlying principles (radioactive filter binding assay and active site-directed competition binding assay). However, it is noteworthy that there was an overlap of kinases targeted by C36 that have been identified via both assays (namely PIM1, TRKA, PDGFRA, MKNK1, MKNK2). This further validates the reliability of the obtained results.

Dose-dependent in vitro ^32^P γ-ATP radiolabeled kinase assays revealed high efficiency of C36 to inhibit LMTK3 at low concentrations (<1 µM), as measured by the phosphorylation of substrate heat shock protein 27 (HSP27) by LMTK3 (Figure 1D). The half maximal inhibitory concentration (IC_50_) of C36 for LMTK3 was approximately 100 nM, as shown by the quantification of the in vitro kinase assay (Figure 1D) [2]. Moreover, as demonstrated by microscale thermophoresis (MST), C36 and C28 displayed comparable affinities to LMTK3 (1.87 ± 0.2 µM and 2.50 ± 0.4 µM, respectively) (Figure 1E). More specifically, this assay measures the movement of fluorescently tagged biomolecules in solution (NHS-647 red dye) through a temperature gradient produced by an infrared (IR) laser [26]. This physical phenomenon is also referred to as “thermophoresis” [27,28]. Since the thermophoretic behavior of a biomolecule depends on its hydration shell, charge, and size [27], the binding of a ligand/drug (in our case C36 and C28) to a molecule of interest (in our case LMTK3) will change the thermophoresis of the molecule of interest [27]. This change in thermophoretic behavior can then be used to analyze the dissociation constant (K_d_) [27].

Following this, we used the interleukin-3 (IL-3)-dependent murine bone marrow-derived cell line FDCP-1 and engineered an LMTK3-transformed clone (FDCP-1/BCR-LMTK3) that relies on the constitutive expression of catalytically active LMTK3 for its survival and proliferation, as described previously [2]. Using this cell-based approach, we assessed the potency of compound C36 and determined the IC_50_ by tracking the cellular viability of FDCP-1 parental and FDCP-1/BRC-LMTK3. As shown in Figure 1F and Figure S1, C36 displayed a higher inhibition of cell viability with FDCP-1/BCR-LMTK3 than the FDCP-1 parental cell line, indicating a C36 inhibition dependent on LMTK3. Taken together, we report the identification of a novel LMTK3 inhibitor (C36), displaying a high in vitro selectivity profile.

### 2.2. Biochemical/Mechanistic Investigation of C36 Binding to LMTK3

**Figure 2:**
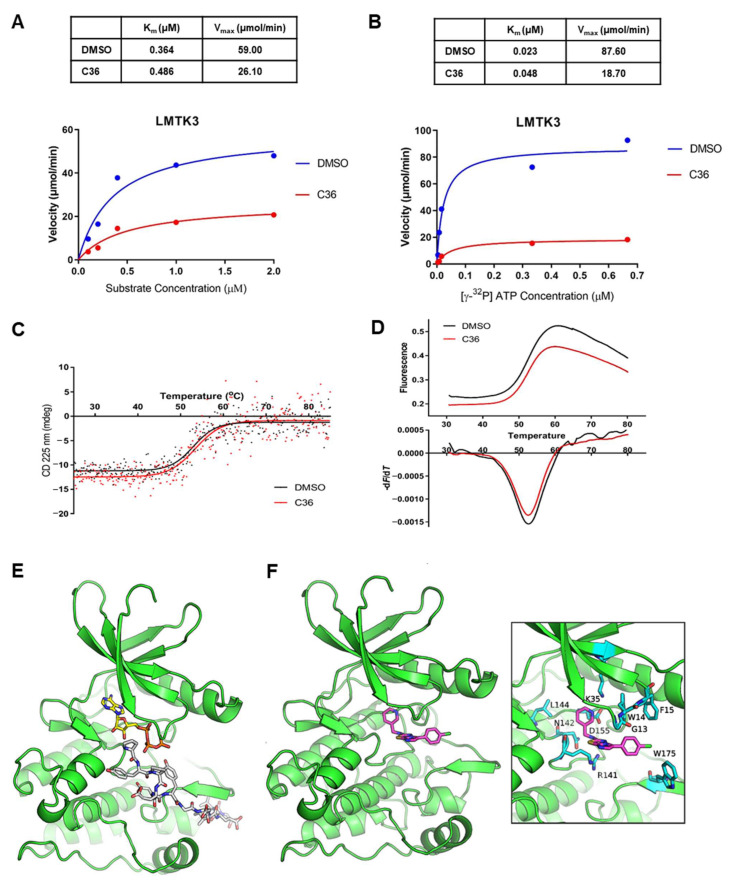
Identification of C36 as a potent inhibitor against LMTK3. (**A**) Kinetic analysis of C36 inhibition with respect to HSP27 concentration (fixed ATP concentration). Kinetic parameters (K_m_ and V_max_) were determined from nonlinear regression fit of the initial reaction rates as a function of HSP27 concentration to the Michaelis–Menten equation using GraphPad Prism 8.01 software (GraphPad Software, Inc., San Diego, CA, USA). (**B**) Kinetic analysis of C36 inhibition as a function of ATP concentration (fixed HSP27 concentration of 0.6 μM). Kinetic parameters (K_m_ and V_max_) were determined from nonlinear regression fit of the initial reaction rates as a function of ATP concentration to the Michaelis–Menten equation using GraphPad Prism 8.01 software. (**C**) Characteristic melting plots obtained from CD spectroscopy for LMTK3 in the absence (DMSO) and presence of inhibitor (C36). (**D**) Characteristic melting curves obtained from thermal shift assay measurements. (**E**) Molecular model of LMTK3 in the active state with bound ATP and a peptide fragment of insulin receptor substrate 2 (IRS2). The kinase domain of LMTK3 is shown with green color, the bound ATP is color-coded with yellow C atoms, and the substrate with grey C atoms; blue is for N, red is for O, yellow is for S, and orange is for P. (**F**) Docked pose of C36 in the active state of ligand-free LMTK3. Inset is a close-up view illustrating residue-specific interactions. C36 is shown with purple C atoms and LMTK3 residues with cyan C atoms.

To investigate the mechanism of action of C36, we examined the effect of increasing HSP27 substrate concentrations on the inhibitory activity of the compound in the presence of constant ATP concentration. Data from the steady-state analysis were fitted to the Michaelis–Menten equation (Figure 2A). Our results from a single technical replicate revealed that the presence of C36 resulted in an increase of K_m_ (0.486 μΜ from 0.364 μM in the absence of C36) with a significantly lower V_max_ (26.1 μmol/min from 59.0 μmol/min in the absence of C36). Next, we investigated the effect of increasing concentrations of ATP at a fixed substrate (HSP27) concentration of 0.6 μM. Similarly, the presence of C36 resulted in a significant increase of the apparent K_m_ (0.048 μM from 0.023 μM in the absence of C36) accompanied by a substantial decrease in V_max_ (18.7 μmol/min from 87.6 μmol/min in the absence of C36, Figure 2B). It is noteworthy to emphasize that these results come from a single replicate and further analysis is required to confirm these data. So far, these results indicate a mixed-type inhibition of LMTK3 by C36, where the inhibitor may overlap with both the ATP- and the substrate HSP27-binding sites without exclusively being a competitive inhibitor of ATP, or the substrate alone.

In addition, we assessed the ability of C36 to bind LMTK3 in solution by monitoring the thermal denaturation of the enzyme in the presence and absence of C36 using a thermal shift assay and circular dichroism (CD) spectroscopy. Both methods displayed a single transition in the thermal melting curves, while the thermal unfolding of LMTK3 was irreversible due to protein aggregation. Our results revealed a minimal influence of the thermal stability of LMTK3 in the presence of C36, with the thermal shift assay indicating a decrease in the T_m_ of LMTK3 by −0.3 ± 0.1 °C (from 52.5 to 52.2 °C in the presence of C36, Figure 2C), and CD spectroscopy showing a small increase of T_m_ by 0.4 ± 0.1 °C in the presence of C36 (from 53.0 to 53.4 °C in the presence of C36, Figure 2D). However, it is important to emphasize that these experiments are not a direct measure of the binding affinity of C36 to LMTK3 due to the intrinsic limitations of these methods [29,30]. Therefore, a change in T_m_ value, whether significant or not, cannot be used to infer binding of our compound to LMTK3. Similarly, one cannot infer that our compound does not bind LMTK3 either. Overall, we conclude that C36 does not have any effect on the thermostability of LMTK3.

Considering that LMTK3 in the absence of ATP and substrate is mainly in the inactive state, the results of the thermal shift assay and CD spectroscopy experiments suggest that C36 has a poor affinity for the inactive state of LMTK3 in solution. Taken together, these results indicate that C36 has no effect in the thermodynamic stability of inactive LMTK3, which contrasts with C28, which displayed a statistically significant stabilization of LMTK3 in the absence of ATP and/or substrate [2].

With the aim to present a putative model of LMTK3 with bound C36 that is in accordance with the above-mentioned results, we prepared a homology model of LMTK3 in the active state and carried out docking of C36. The inactive state of LMTK3 remains the only available X-ray structure where the ATP-binding site is occluded by the DYG-motif Tyr^314^ [2]. Considering the potentially low affinity of C36 for the LMTK3 inactive state and the relatively high sequence identity between the kinase domain of insulin receptor (IRK) and LMTK3 (37%) (Figure S3), we thus employed the X-ray structure of IRK in complex with ATP and a peptidic substrate (PDB ID: 3bu5) [31] as a template for modelling of LMTK3 in the active state. Our docking results suggest that C36 could bind adjacent to the ATP-binding site of LMTK3 and interact with the substrate as well (Figure 2E,F). This binding mode is also in accordance with the mixed-type inhibition profile of C36, as observed in the kinetic analysis.

### 2.3. C36 Exhibits Potent Anticancer Activity in Different Human Cancer Cell Lines

**Figure 3:**
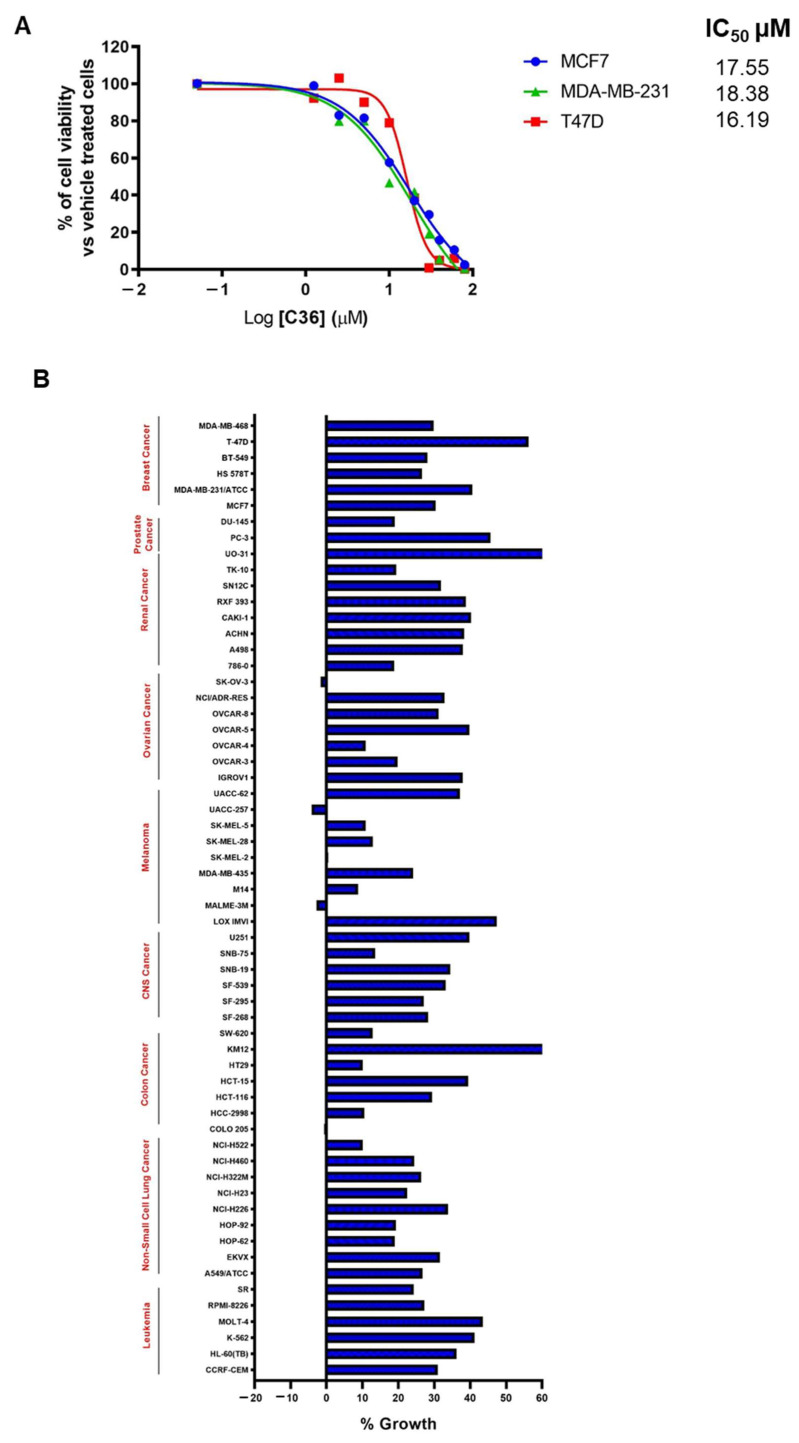
C36 impairs the viability of various human cancer cell lines. (**A**) Viability of BC cell lines treated with increasing concentrations of C36 for 72 h. The IC_50_ values are means from three independent experiments. (**B**) One-dose screening of C36 (10 µM; 24 h) on the NCI-60 panel of tumor cell lines. The percent growth of C36-treated cells is shown. Negative values represent lethality.

We then investigated the potential use of C36 as an anticancer strategy by examining the viability of various BC cell lines in the presence of increasing concentrations of C36. As shown in Figure 3A, C36 was able to inhibit the growth of MCF7, T47D, and MDA-MB-231 BC cells, with IC_50_ values ranging from 16.19 µM to 18.38 µM. Following this, we submitted C36 to the Developmental Therapeutics Program (DTP) of the National Cancer Institute (NCI) and screened it against a panel of 60 human cancer cell lines [32]. Interestingly, our results showed that at a 10 µM dose, C36 inhibited all cancer cell lines by >40% (Figure 3B).

**Figure 4:**
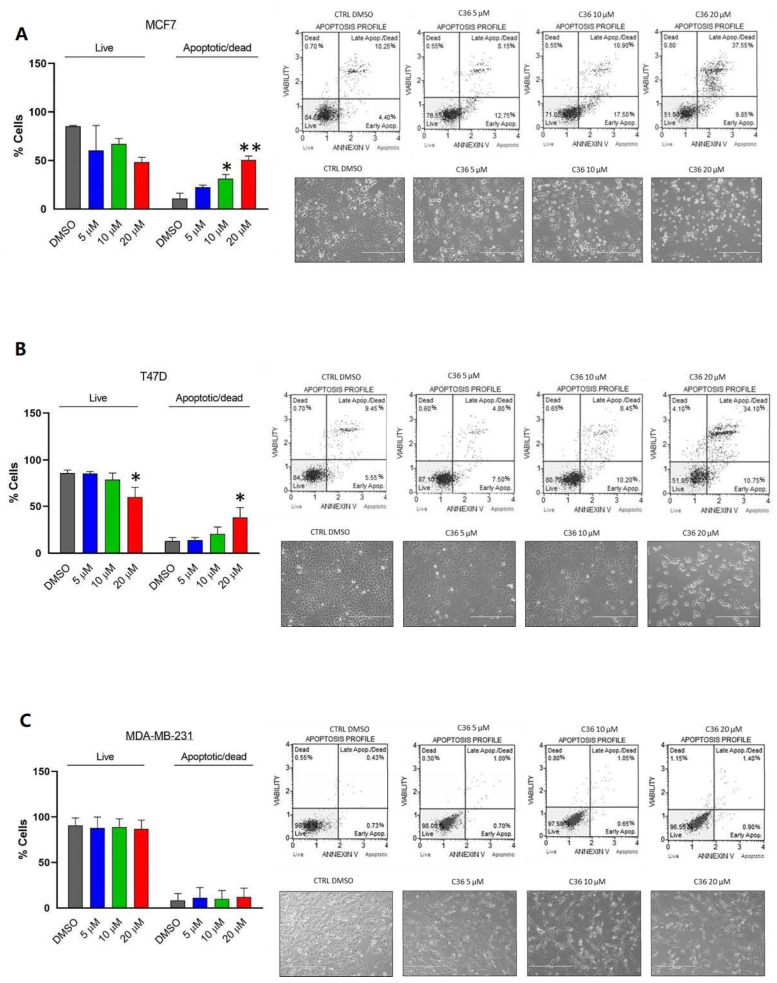
Apoptotic effect of C36 on different human breast cancer cell lines. MCF7 (**A**), T47D (**B**), and MDA-MB-231 (**C**) were treated with increasing concentrations of C36 for 96 h. The percentages of apoptotic and dead cells were analyzed by Annexin V and 7-AAD staining. Results are expressed as means ± SEM; * *p* < 0.05, ** *p* < 0.01.

Finally, we investigated the apoptotic properties of C36 in the aforementioned BC cell lines using annexin V and 7-AAD (7-amino-actinomycin D) staining. As shown in Figure 4A,B, following treatment for 96 h C36 exhibited an apoptotic effect at 20 μM in MCF7 and T47D BC cell lines, respectively. For MCF7 cells, apoptotic effects of C36 were also detected at 10 μM (Figure 4A). Lastly, no apoptosis was detected in MDA-MB-231 BC cell line, even when treated with 20 μM C36 (Figure 4C). Specifically, MCF7 and T47D cell lines displayed late apoptotic effects after 20 µM C36 treatment (Figure S4). Taken together, these results show that different BC cell lines display sensitivity to C36 treatment in terms of proliferation and apoptosis.

### 2.4. Pharmacological Properties of C36

The metabolic stability of C36 was also analyzed by incubating this drug with mouse hepatic microsomes (1 µM initial concentration, 0.25 mg protein/mL). Our results show that C36 was metabolized, relatively quickly, with a half-life value of 22 min and a high intrinsic clearance (Clint) value of 132 µL/min/mg (Figure S5A). This microsomal stability assay also produced two detectable putative metabolites, the most abundant of which is shown in Figure S5B. In addition, C36 showed low passive permeability in Caco-2 monolayer experiments (A > B; Papp of 5.1 × ≤10^−6^ cm/s), which could limit its absorption in in vivo studies, although its efflux ratio of 2.1 is not excessive (Figure S5A).

## 3. Discussion

Despite the increasingly established role of LMTK3 in several cancer types and its central role in a number of well-described signaling pathways [8,10,15,20], currently there are no drugs in clinical trials targeting this oncogenic kinase. Here, we report a new tool compound, namely C36, which exhibits anticancer activity against a variety of cancer cell lines that is at least partly mediated by LMTK3 [2]. Based on our data, we propose that C36 not only competes with the LMTK3 ATP-binding site but also with the substrate-binding site in the kinase active state. Our molecular model suggests that C36 can interact with the ATP-binding site of LMTK3 and with the substrate as well, confirming the mixed-type inhibitory profile of C36. It is well established that most protein kinase inhibitors in clinical development mainly target the highly conserved ATP-binding site and thus are likely to have many off-target effects against kinases unrelated to diseases. Therefore, inhibitors like C36 that also possess competitive properties towards the kinase’s substrates are considered more selective and are expected to be promising therapeutic agents [33].

Recently, we reported the first tool compound (C28) against LMTK3 that displays anticancer activity in a variety of cancer cell lines and in vivo BC mouse models [2]. C36 and C28 displayed comparable affinities to LMTK3, as shown by MST. Importantly, C36 has a higher selectivity to purified human kinases when compared to C28 highlighting it as a promising candidate for drug development against LMTK3. Moreover, C36 demonstrates a strong antiproliferative effect against different cancer cell lines. Based on our results, C36 also exhibited apoptotic effects against BC cell lines (MCF7 and T47D) following a longer treatment exposure (96 h) and higher drug concentration (20 μM) than required to induce apoptosis following C28 treatment (72 h and 10 μM) [2].

The analysis of C36 metabolic stability was performed by incubating this drug with mouse hepatic microsomes. The results indicate C36 was metabolized relatively quickly. This cell-based system is not the ideal indicator for mouse in vivo studies; however, it is a commonly used steppingstone that can correlate well with liver microsomal stability in human and in vivo activity in mice [34,35,36]. Moreover, given the short half-life of C36, in vivo oral treatment at 0.25 mg protein/ml would be attainable only for a short period of time. In addition, given the short half-life of the drug (22 min) and its low passive permeability (A > B; Papp of 5.1 × ≤ 10^−6^ cm/s), work is currently underway, focusing on the design of C36 analogues and testing their effects in xenograft models of BC. Additional testing in other types of non-cancerous cell lines will provide further validation regarding C36 selectivity against cancer cell lines.

Future work will focus on studying the specific molecular interactions between C36 and the kinase domain of LMTK3 by performing co-crystallization experiments. Investigating which amino acid residues are involved will likely shed light onto the mechanism of action of C36, furthering our knowledge. From our preliminary data presented in this manuscript, C36 decreases the rate of HSP27 phosphorylation by LMTK3 kinase domain. Therefore, additional experiments might include co-crystallizing the entire complex (LMTK3-HSP27-C36) in order to understand, in more detail, the specific molecular interactions involved. Additional C36 analogues are also being synthesized to improve the binding affinity of the drug to LMTK3 and to increase its inhibitory properties against LMTK3 activity both in vitro (kinetic analysis) and in breast cancer cell lines.

Since LMTK3 has been shown to have a fundamental role in breast cancer progression and since there are no current drugs available targeting this oncogenic kinase, LMTK3 inhibitors could represent a valid alternative treatment to breast cancer patients. More specifically, LMTK3 inhibitors could be combined alongside aromatase inhibitors (AIs) as an alternative to treatment with CDK4/6 inhibitors to improve patient outcome in estrogen receptor positive (ER+) BC [37,38]. Likewise, given the aberrant expression of LMTK3 in triple negative breast cancer (TNBC) and studies showing that LMTK3 inhibition results in inhibition of TNBC cell proliferation, migration and invasion [14,16,17], the use of LMTK3 inhibitors could have beneficial effects for this clinically unmet category of BC patients.

In addition, since the mechanism of emergence of endocrine and chemotherapy resistance in BC remains largely unclear [2], there is a need to treat these patients in a more focused way. Based on our previous studies, inhibition of LMTK3 appears to be implicated in re-sensitization of cells to tamoxifen and doxorubicin treatment [8,12,13,14]. Consequently, an LMTK3 drug could be used alongside established therapies to increase the sensitivity of tumors to treatment and/or potentially overcome resistance. Ultimately, this paper provides a steppingstone for the development and optimization of oral LMTK3 inhibitors, including C36, for use in clinical applications, either as a monotherapy or as a combination therapy in breast cancer.

Finally, immunotherapy has become an established mainstay in cancer treatment and new drugs are being promptly developed for use in clinical settings [39]. Monoclonal antibodies (mAb), Ab-drug conjugates (ADCs), and cancer vaccines all represent different types of immunotherapies used in the treatment of BC [39] and other cancers. Currently, there are no immunotherapy programs specifically targeting LMTK3 in BC. However, the combination of immuno-therapeutic drugs (immune checkpoint inhibitor atezolizumab (Tecentriq^®^, Genentech, San Francisco, CA, USA)) and chemotherapeutic agents (nabPTX (Abraxane^®^, Celgene, Summit, NJ, USA)) has already been applied for the treatment of TNBC [34]. Based on this, novel LMTK3 inhibitors may be used in combination with immunotherapy and chemotherapy drugs [40] to improve the treatment of BC.

## 4. Materials and Methods

### 4.1. Cell Lines

MCF7, T47D, and MDA-MB-231 cell lines were purchased from ATCC. MCF7 and MDA-MB-231 were maintained in low glucose DMEM (Sigma Aldrich, St. Louis, MO, USA, #D6046-500ML) supplemented with 10% FBS (Sigma Aldrich, #F7524-500ML) and 1% Penicillin/Streptomycin (Sigma Aldrich, #P0781-100ML). T47D cell line was maintained in RPMI-1640 medium (Sigma Aldrich, #R5886-500ML) supplemented with 10% FBS (Sigma Aldrich, #F7524-500ML) and 1% L-glutamine/Penicillin/Streptomycin solution (Sigma Aldrich, #G1146-100ML).

### 4.2. Cell Death and Apoptosis

Cells were treated with increasing concentrations of C36 for 96 h. After collection, cells were stained with the Muse Annexin V Dead Cell Kit according to the manufacturer’s protocol (Millipore, Burlington, MA, USA, #MCH100105). Cells were then analyzed using the Muse Cell Analyzer (Millipore). Statistical analysis was performed with GraphPad Prism 8.0.1 software. In particular, one-way ANOVA analysis of variance with Dunnet post-hoc test for multiple comparison was performed. Statistical significance refers to the sample compared to the control (DMSO). *p* values < 0.05 were considered to be statistically significant.

### 4.3. Cell Viability Assay

Cell viability assay was performed as previously described [41]. Mammalian cells were cultured at 3000 cells/well in 96-well plates (Corning, Corning, NY, USA, #3603). FDCP1 and LMTK3-transformed FDCP1 cells were plated at 5000 cells/well in 384-well plates (Aurora Biotechnologies, Poway, CA, USA, cat. no. 2030-10200). Cell viability was assessed using the CellTiter-Glo luminescent cell viability assay (Promega, Madison, WI, USA, #G7572), as previously described [14]. Data analysis was performed with GraphPad Prism 8.01 software. In particular, we performed a nonlinear regression (curve fit) analysis by using the “dose–response–inhibition” model (log(inhibitor) vs. response–variable slope (four parameters)) to calculate the IC_50_ values as previously described [2].

### 4.4. In Vitro Kinase Assay

^32^P γ-ATP in vitro kinase assays were performed in-house, as we have previously described [40]. The intensities of the bands on the autoradiograms have been quantified using ImageJ 1.53t software (Wayne Rasband and contributors, National Institutes of Health, Madison, WI, USA) and normalized to total protein levels based on Coomassie Blue stained membranes. DMSO has been used as a control.

### 4.5. Kinase Inhibitor Competition Binding Assay

The selectivity profiling of C36 kinase inhibitor at 5 µM was analyzed using DiscoverX KINOMEscan competition binding assay against a panel of 403 kinases [25]. The KINOMEscan screening platform uses a novel active site-directed competition binding assay to measure interactions between a specific compound and approximately 400 kinases in a quantitative manner. The KINOMEscan assay does not require ATP and therefore reports true thermodynamic interaction affinities, instead of IC_50_ values, which usually depend on the ATP concentration. In particular, “hits” are detected by measuring the amount of kinase captured in test versus control samples by using qPCR, which is a method that detects the associated DNA label [25].

### 4.6. Microscale Thermophoresis (MST)

Purified LMTK3 protein was labelled with an NHS-647 red dye (NanoTemper Technologies, München, Germany), following the manufacturer’s protocol. Serial dilutions of C36 (200 µΜ–0.61 nM) and C28 (200 µΜ–3.05 nM) in MST buffer (50 mM Tris pH 7.4, 150 mM NaCl, 10 mM MgCl2, 0.05% Tween 20, 2% DMSO) were mixed with 50 nM NHS-647-labeled LMTK3 and loaded into standard glass capillaries (Monolith NT.115 Capillaries, NanoTemper Technologies). The final DMSO concentration was kept below 5%, as indicated by Ref. [26]. Thermophoresis analysis was performed over 20 sec on a Monolith NT.115 instrument (80% LED, 60% MST power) at 24 °C. The MST curves were fitted using NT Analysis software (NanoTemper Technologies) to obtain K_d_ values for binding.

### 4.7. Thermal Shift Assay 

A thermal shift assay was performed using Roche LightCycler 96 real-time polymerase chain reaction (RT-PCR) instrument, with excitation and emission wavelengths set to 533 and 572 nm, respectively. Solutions comprising 16 µL of 5.4 µM LMTK3 in 200 mM tris buffer (pH 8.0), 200 mM NaCl, and 4 µL of 50× SYPRO orange (Sigma-Aldrich, St. Louis, MO, USA) and 0.2 µL of either dimethyl sulfoxide (DMSO) or C36 in DMSO (final concentration of 10 µΜ C36, 1% (*v*/*v*) DMSO, 4.3 µΜ LMTK3, and 10× SYPRO orange). The temperature range spanned from 25 °C to 80 °C at a scan rate of 1 °C/min. Data analysis was performed in LightCycler 96 (v1.1, Roche, Mannheim, Germany) software using the melting curve analysis, and T_m_ values were determined as the first negative derivative of the fluorescence with respect to the temperature.

### 4.8. CD Spectroscopy

CD spectroscopy was performed using a Jasco J-715 instrument (Jasco, Tokyo, Japan) equipped with a PTC-348 temperature control unit. Temperature increased from 20 °C to 90 °C at an increment of 1 °C/min, and data points were acquired every 0.2 °C by monitoring a wavelength of 230 nm. For thermal stability experiments, LMTK3 samples of 5.4 µΜ in 200 mM tris buffer (pH 8.0) and 200 mM NaCl were treated with either DMSO 0.4% (*v*/*v*) or 8.3 µΜ C36 in DMSO (0.4%) to a total volume of 120 µL in 0.1 cm cuvettes. Data analysis was performed in GraphPad Prism 8.01 software by fitting data in the transition region to a Boltzmann sigmoidal. Apparent T_m_ values were determined as the point at which the transition was 50% complete.

### 4.9. Molecular Modelling of LMTK3 with Bound C36

The homology model of LMTK3 in the active state and the X-ray structure of the kinase domain of human insulin receptor (IRK), in complex with ATP and a peptidic-substrate (PDB ID: 3bu5) [31], were prepared using Modeller v9.24 (University of California San Francisco, San Francisco, CA, USA) [42]. The alignment is shown in the Supplementary Figure S3. The model with the lowest DOPE score was employed for docking of C36 using AutoDock v4.2 (The Scripps Research Institute, La Jolla, CA, USA) [43] with default parameters, except for the number of docking rounds set to 100, and number of energy evaluations set to 10 million. Results were clustered with a *rmsd* tolerance of 2.0 Å (Supplementary Figure S6), and the top-ranked pose was selected as the putative bound conformation of C36 in the active state of LMTK3 (Figure 2F). The model of LMTK3 in complex with ATP and substrate (Figure 2E) was generated by superimposing the bound ATP and peptide substrate from the insulin receptor X-ray structure onto the model of active LMTK3, and after energy minimization with positional restraints on all Cα atoms (10 kcal × mol^−1^ × Å^−2^) using AMBER v16 (UCSF, San Francisco, CA, USA) [43].

### 4.10. Caco-2 Permeability Assay

The bi-directional Caco-2 cell permeability assay was performed as described in the BioFocus DPI Ltd. Standard Operating Procedure, ADME-SOP-49. Caco-2 cells (ECACC) were seeded onto 24-well Transwell plates at 2 × 10^5^ cells per well and used in confluent monolayers after a 21-day culture at 37 °C under 5% CO_2_. Test and control compounds (propranolol, vinblastine), prepared in DMSO, were added (10 µM, 0.1% DMSO final, n = 2) to donor compartments of the Transwell plate assembly in assay buffer (Hanks balanced salt solution supplemented with 25 mM HEPES, adjusted to pH 7.4) for both apical to basolateral (A > B) and basolateral to apical (B > A) measurements. Incubations were performed at 37 °C, with samples removed from both donor and acceptor chambers at T = 0 and 1 h and compound analyzed by mass spectrometry (LC-MS/MS) including an analytical internal standard. Apparent permeability (P_app_) values were determined from the relationship:

P_app_ = [Compound_Acceptor T=end_] × V_Acceptor_/([Compound_Donor T=0_] × V_Donor_)/incubation time × V_Donor_/Area × 60 × 10^−6^ cm/s.

V is the volume of each Transwell compartment (apical 125 µL, basolateral 600 µL), and concentrations are the relative MS responses for compound (normalized to internal standard) in the donor chamber before incubation and acceptor chamber at the end of the incubation.

Area = area of cells exposed for drug transfer (0.33 cm^2^).

Efflux ratios (P_app_ B > A/P_app_ A > B) were calculated for each compound from the mean P_app_ values in each direction. A finding of good permeability B > A, but poor permeability A > B, suggests that a compound is a substrate for an efflux transporter, such as P-glycoprotein.

Lucifer Yellow (LY) was added to the apical buffer in all wells to assess viability of the cell layer. As LY cannot freely permeate lipophilic barriers, a high degree of LY transport indicates poor integrity of the cell layer and wells with a LY P_app_ > 10 × 10^−6^ cm/s were rejected. Note that an integrity failure in one well does not affect the validity of other wells on the plate.

Compound recovery from the wells was determined from MS responses (normalized to internal standard) in donor and acceptor chambers at the end of incubation compared to response in the donor chamber pre-incubation. Recoveries < 50% suggest compound solubility, stability, or binding issues in the assay, which may reduce the reliability of a result.

### 4.11. Compound Stability in Mouse Hepatic Microsomes

Microsomal stability assays were performed as described in the BioFocus DPI Ltd. Standard Operating Procedure, ADME-SOP-84, using pooled hepatic microsomes from mouse (Xenotech/1210302, Kansas City, KS, USA). Test and control compounds (dextromethorphan and midazolam), prepared in DMSO, were incubated at an initial concentration of 1 µM (0.25% DMSO final, n = 2) with microsomes (0.25 mg protein/ml) at 37 °C in the presence and absence of the cofactor, NADPH (1 mM). Aliquots were removed at 0, 5, 10, 20, and 40 min for termination of reactions and compound extraction with acetonitrile containing an analytical internal standard. Samples were centrifuged and the supernatant fractions were analyzed for parent compound by mass spectrometry (LC-MS/MS).

The amount of compound remaining (expressed as %) was determined from the MS response in each sample relative to that in the T = 0 samples (normalized for internal standard).

Ln plots of the % remaining were used to determine the half-life for compound disappearance using the relationship: t_1/2_ (min) = −0.693/λ, where λ is the slope of the Ln % remaining vs. time curve.

The in vitro intrinsic clearance (CLint) (µL/min/mg microsomal protein) was calculated using the formula: CLint = 0.693 × 1/t½ (min) × (1/mg of microsomal protein/ml) × 1000.

### 4.12. NCI-60 Human Tumor Cell Line Screen

The NCI-60 panel of tumor cell lines utilizes a variety of different cancerous cell lines to identify and characterize novel compounds that inhibit the growth or exert a lethal effect on these tumor cells. This screen encompasses 60 cell lines from leukemia, melanoma, and cancers of the colon, brain, ovary, lung, prostate, breast, and kidney. In our case, 60 different cell lines were treated with 10 µM C36 for 24 h. Following this, growth inhibition and lethality were measured [32].

## Data Availability

All data needed to evaluate the conclusions in the paper are present in the paper and/or the Supplementary Materials. Additional data related to this paper may be requested from the authors.

## Supplementary Materials

The following supporting information can be downloaded at: https://www.mdpi.com/article/10.3390/ijms24010865/s1.
