# Supplementary material for: The Inhibitory Properties of a Novel, Selective LMTK3 Kinase Inhibitor
Source: Int J Mol Sci. 2023 Jan 3;24(1):865. doi: 10.3390/ijms24010865 (PMC9821308; doi:10.3390/ijms24010865)
Supplement: Supplementary file 1 [file ijms-24-00865-s001.zip › ijms-1957734-supplementary.pdf]

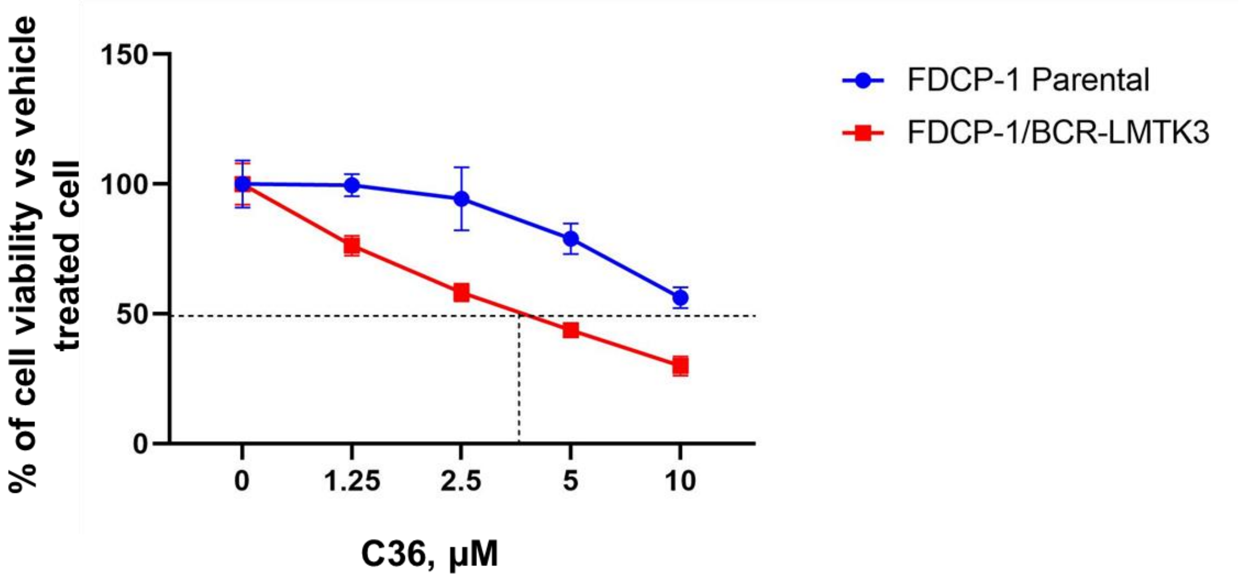

Figure S1: IC50 values for C36 in FDCP1 and FDCP1-LMTK3 cell lines. Error bars represent the means ± SD from three independent experiments.

Fig. S1

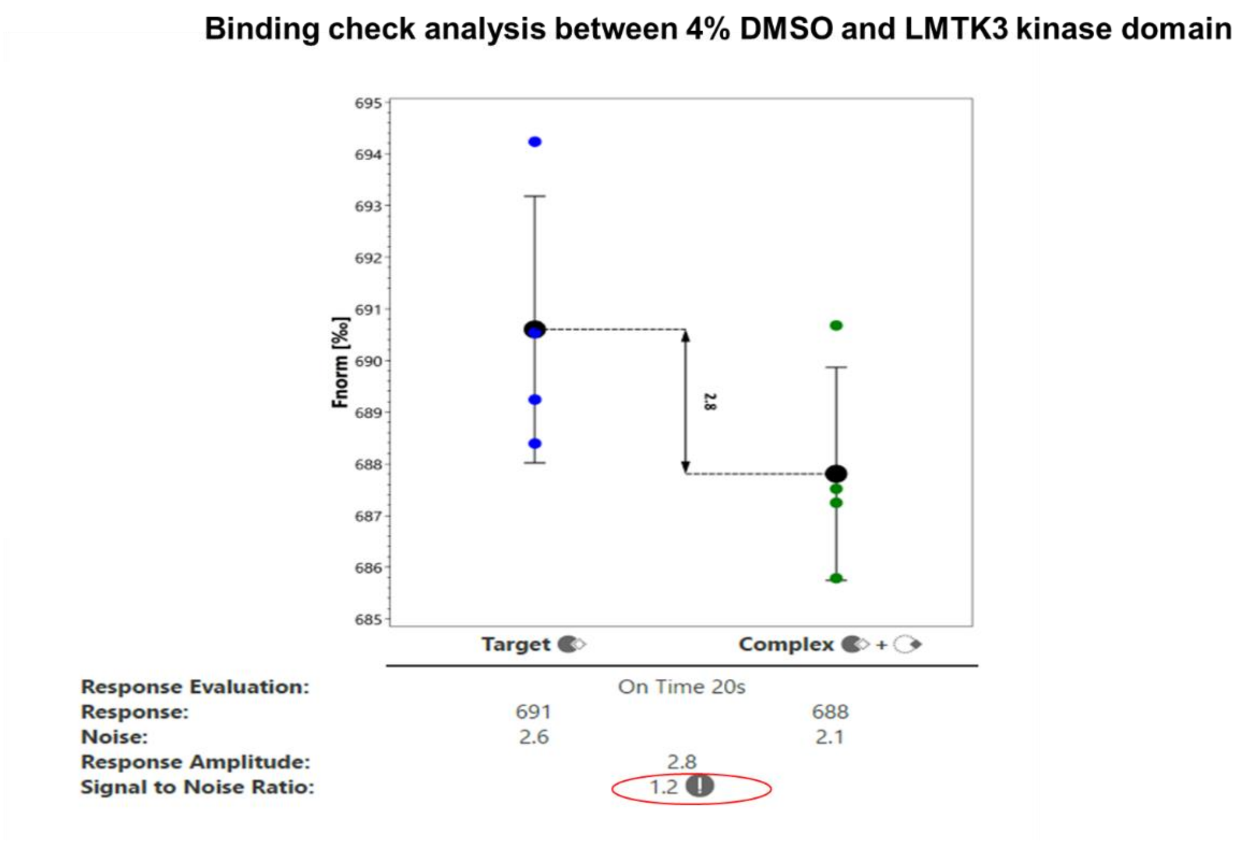

Figure S2: Binding check analysis between DMSO and LMTK3 kinase domain. The signal to noise ratio is used to evaluate the quality of the binding data. It is defined as the response amplitude di-vided by the noise of the measurement. A signal to noise ratio of more than 5 is desirable, while more than 12 reflects an excellent binding assay (NT Analysis software (NanoTemper Technologies).

Fig. S2

```
Query: LMTK3 (PDB ID: 6SEQ)                                Subject: IRK (PDB ID: 3BU5)
Range 1: 13 to 277                                      Alignment statistics for match #1
Score          Expect      Method                       Identities     Positives     Gaps
174 bits(441)1e-47Compositional matrix adjust.          102/277(37%) 159/277(57%) 16/277(5%)

Query  128  LSRQHLSYLQEIGSGWFGKVILG---EIFSDYTPAQVVVKELRASAGPLEQRKFISEAQP  184
            +SR+ ++ L+E+G G FG V  G   +I       +V VK +  SA   E+ +F++EA
Sbjct  13   VSREKITLLRELGQGSFGMVYEGNARDIIKGEAETRVAVKTVNESASLRERIEFLNEASV  72

Query  185  YRSLQHPNVLQCLGLCVETLPFLLIMEFCQLGDLKRYLRAQRPPEGLSPELPPRDLRTLQ  244
             +     +V++ LG+  +  P L++ME    GDLK YLR+ RP    +P  PP  L+ +
Sbjct  73   MKGFTCHHVVRLLGVVSKGQPTLVVMELMAHGDLKSYLRSLRPEAENNPGRPPPTLQEMI  132

Query  245  RMGLEIARGLAHLHSHNYVHSDLALRNCLLTSDLTVRIGDYGLAHSNYKEDYYLTPERLW  304
            +M  EIA G+A+L++  +VH DLA RNC++  D TV+IGD+G+    Y+ DYY    +
Sbjct  133  QMAAEIADGMAYLNAKKFVHRDLAARNCMVAHDFTVKIGDFGMTRDIYETDYYRKGGKGL  192

Query  305  IPLRWAAPELLGELHGTFMVVDQSRESNIWSLGVTLWELFEFGAQPYRHLSDEEVLAFVV  364
            +P+RW APE L +  G F     +  S++WS GV LWE+     QPY+ LS+E+VL FV+
Sbjct  193  LPVRWMAPESLKD--GVF-----TTSSDMWSFGVVLWEITSLAEQPYQGLSNEQVLKFVM  245

Query  365  RQQHVKLARPRLKLPYADYWYDILQSCWR-PPAQRPS  400
               +  L +P    P  +   D+++ CW+  P  RP+
Sbjct  246  DGGY--LDQPD-NCP--ERVTDLMRMCWQFNPKMRPT  277
```

```
CLUSTAL O(1.2.4) multiple sequence alignment
>6SEQ_1|Chain A|Serine/threonine-protein kinase LMTK3|Homo sapiens (9606)
>3BU5_1|Chain A|insulin receptor subunit beta|Homo sapiens (9606)
1: 6SEQ_1|Chain  100.00   36.78
2: 3BU5_1|Chain   36.78  100.00

6SEQ_1|Chain      LSRQHLSYLQEIGSGWFGKVILGEI---FSDYTPAQVVVKELRASAGPLEQRKFISEAQP
3BU5_1|Chain      VSREKITLLRELGQGSFGMVYEGNARDIIKGEAETRVAVKTVNESASLRERIEFLNEASV
                  :**:::: *:*:*.* ** *  *:    :.. : ::*.** :. **.  *: :*:.**.

6SEQ_1|Chain      YRSLQHPNVLQCLGLCVETLPFLLIMEFCQLGDLKRYLRAQRPPEGLSPELPPRDLRTLQ
3BU5_1|Chain      MKGFTCHHVVRLLGVVSKGQPTLVVMELMAHGDLKSYLRSLRPEAENNPGRPPPTLQEMI
                   :.:   :*:: **:  :  * *::**:   **** ***: **    .*  **  *: :

6SEQ_1|Chain      RMGLEIARGLAHLHSHNYVHSDLALRNCLLTSDLTVRIGDYGLAHSNYKEDYYLTPERLW
3BU5_1|Chain      QMAAEIADGMAYLNAKKFVHRDLAARNCMVAHDFTVKIGDFGMTRDIYETDYYRKGGKGL
                  :*. *** *:*:*:::::** *** ***::: *:**:***:*:::. *: ***  .  :

6SEQ_1|Chain      IPLRWAAPELLGELHGTFMVVDQSRESNIWSLGVTLWELFEFGAQPYRHLSDEEVLAFVV
3BU5_1|Chain      LPVRWMAPESLKDG--VF-----TTSSDMWSFGVVLWEITSLAEQPYQGLSNEQVLKFVM
                  :*:** *** * :   .*     : .*::**:**.***: .:. ***: **:*:** **:

6SEQ_1|Chain      RQQHVKLARPRLKLPYADYWYDILQSCWRPPA-QRPS
3BU5_1|Chain      DGGYLDQPD-----NCPERVTDLMRMCWQFNPNMRPT
                     ::.           :   *::: **:      **:
```

Figure S3: Sequence alignment of LMTK3 and IRK.

Fig. S3

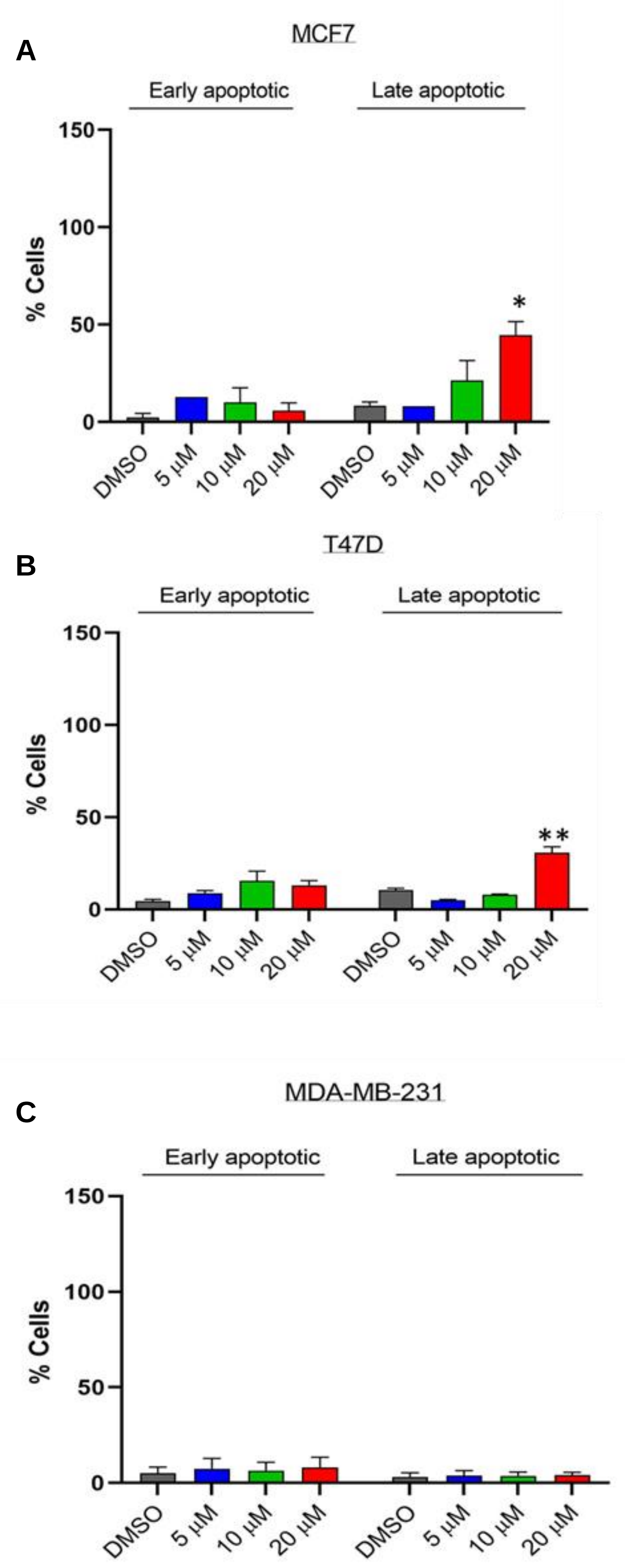

Figure S4: Apoptotic effect of C36 on different human breast cancer cell lines. MCF7 (A), T47D (B) and MDA-MB-231 (C) were treated with increasing concentrations of C36 for 96 h. The percentages of early apoptotic and late apoptotic cells were analyzed by Annexin V and 7-AAD staining. Results are expressed as means ± SEM; * $p < 0.05$, ** $p < 0.01$.

Fig. S4

A

Metabolic Stability		Caco-2 Permeability Assay	
Half-life (minutes)	Intrinsic clearance (µL/min/mg)	Passive permeability (cm/sec)	Efflux ratio
22	132	5.1 x ≤10^{-6}	2.1

B

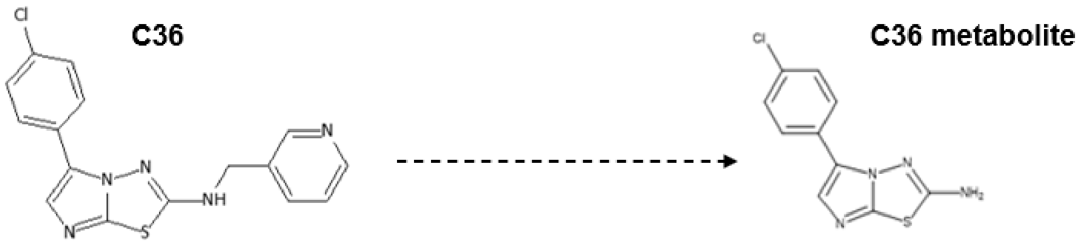

Figure S5: Pharmacological properties of C36.

Fig. S5

```
      Number of distinct conformational clusters found = 12,  out of 100 runs,
      Using an rmsd-tolerance of 2.0 A

      CLUSTERING HISTOGRAM
________________________________________________________________________________
     |           |     |           |     |
Clus | Lowest    | Run | Mean      | Num | Histogram
-ter | Binding   |     | Binding   | in  |
Rank | Energy    |     | Energy    | Clus|    5    10   15   20   25   30   35
_____|___________|_____|___________|_____|____:____|____:____|____:____|____:___
   1 |     -9.04 |  82 |     -8.76 |  51 |########################################
   2 |     -8.91 |  12 |     -8.77 |  13 |#############
   3 |     -8.16 |  18 |     -8.11 |   6 |######
   4 |     -8.11 |  84 |     -8.09 |   8 |########
   5 |     -8.06 |  94 |     -7.97 |   4 |####
   6 |     -8.04 |  41 |     -8.04 |   1 |#
   7 |     -7.87 |  30 |     -7.79 |   8 |########
   8 |     -7.68 |  28 |     -7.68 |   1 |#
   9 |     -7.63 |  90 |     -7.62 |   5 |#####
  10 |     -7.50 |   7 |     -7.50 |   1 |#
  11 |     -7.46 |  79 |     -7.46 |   1 |#
  12 |     -7.46 |  83 |     -7.46 |   1 |#
_____|___________|_____|___________|_____|______________________________________

Number of multi-member conformational clusters found = 7, out of 100 runs.
```

```
MODEL       82
USER    Run = 82
USER    Cluster Rank = 1
USER    Number of conformations in this cluster = 51
USER
USER    RMSD from reference structure       = 72.756 A
USER
USER    Estimated Free Energy of Binding    =   -9.04 kcal/mol  [=(1)+(2)+(3)-(4)]
USER    Estimated Inhibition Constant, Ki   =  236.15 nM (nanomolar)  [Temperature = 298.15 K]
USER
USER    (1) Final Intermolecular Energy     =   -9.94 kcal/mol
USER        vdW + Hbond + desolv Energy     =   -9.64 kcal/mol
USER        Electrostatic Energy            =   -0.29 kcal/mol
USER    (2) Final Total Internal Energy     =   -0.11 kcal/mol
USER    (3) Torsional Free Energy           =   +0.89 kcal/mol
USER    (4) Unbound System's Energy  [=(2)] =   -0.11 kcal/mol
USER
```

Figure S6: Docking results of C36 to the model of active LMTK3.

Fig. S6
